# Patient attitudes and beliefs associated with self-referral to physical therapy for musculoskeletal complaints: a qualitative study

**DOI:** 10.1186/s12913-022-08989-x

**Published:** 2023-01-25

**Authors:** Noor Alshareef, Melanie Cozad, Mark Macauda, Jan Ostermann, Charles Thigpen

**Affiliations:** 1 Department of Health Services Policy and Management, Arnold School of Public Health, University of South Carolina, 921 Assembly St, Columbia, SC 29208 USA; 2 Department of Health Services Research and Administration, Collage of Public Health, University of Nebraska Medical Center, 984350 Nebraska Medical Center, Omaha, NE 68198 USA; 3 Department of Health Promotion, Education, and Behavior, Arnold School of Public Health, University of South Carolina, 921 Assembly St, Columbia, SC 29208 USA; 4 ATI Physical Therapy, 200 Patewood Dr, Greenville, SC 29615 USA

**Keywords:** Non-pharmacologic treatment, Choice, Direct access

## Abstract

**Background:**

Non-pharmacologic treatments such as physical therapy (PT) are advocated for musculoskeletal pain. Early access to PT through self-referral has been shown to decrease costs and improve outcomes. Although self-referral is permitted in most U.S. states and supported by some health insurance plans, patients’ utilization of self-referral remains low.

**Objective:**

To identify factors, beyond legislative policies and health insurance, associated with patients’ decisions to access physical therapy through self-referral or provider-referral.

**Methods:**

We recruited 26 females and 6 males whose employer-sponsored insurance benefits included financial incentives for self-referral to physical therapy. Between August 2017 and March 2018, participants completed semi-structured interviews about their beliefs about physical therapy and reasons for choosing self-referral (15 participants) or provider referral (17 participants) for accessing physical therapy. Grounded theory approach was employed to identify themes in the data.

**Results:**

Patients selecting self-referral reported major thematic differences compared to the provider-referral patients including knowledge of the direct access program, attitudes and beliefs about physical therapy and pharmacologic treatment, and prior experiences with physical therapy. Self-referral patients were aware that their plan benefits included reduced cost for self-referral and felt confident in selecting that pathway. They also had negative beliefs about the effectiveness of pharmacological treatments and surgery, and previously had positive direct or indirect experiences with physical therapy.

**Conclusion:**

Knowledge of the ability to self-refer, attitudes and beliefs about treatment, and prior experience with physical therapy were associated with self-referral to physical therapy. Interventions aimed at improving knowledge and changing attitudes toward self-referral to physical therapy to increase utilization appear warranted.

## Introduction

Non-pharmacologic treatments such as physical therapy are advocated for musculoskeletal pain to decrease opioid use [1]. Early access to physical therapy via self-referral has been shown to decrease health care costs and improve patient outcomes [2–5]. Self-referral (direct access) refers to a treatment pathway in which patients are evaluated and treated by a physical therapist without receiving a prior physician consultation [6]. Self-referral is safe, efficient, and cost-effective; however, utilization remains low and is estimated at around 6% in privately insured populations [7–9].

Within the United States (U.S.), many factors may influence a patient’s decision to access physical therapy via self-referral. Once a patient identifies a need for physical therapy, the choice of treatment pathway (self-referral or provider referral) may be constrained by state-level legislation, institutional-level policies, or individual-level characteristics. Self-referral laws and regulations about access to physical therapy services for treatment and evaluation vary across states. Some states allow for unrestricted self-referral, others allow for self-referral with provisions; yet others limit patient self-referral [10]. Even if a patient resides in a state that offers some form of self-referral, the choice of self-referral to physical therapy might be restricted because of institutional-level policies developed by insurance providers, and/or healthcare organizations’ management policies [7, 11, 12].

Independent of state and organizational barriers, self-referral to physical therapy may also be influenced by patient-level determinants. Research has identified multiple patient-specific determinants that influence how a patient accesses physical therapy, including condition-related characteristics [2, 3, 13, 14], past physical therapy treatment experiences [13, 14], sociodemographic characteristics [2, 11, 13–15], knowledge of the ability to self-refer [7, 16], attitudes to and beliefs about access [15, 16], and the geographical location of the physical therapy practice [3].

Evidence on the clinical and cost-effectiveness of “self-Webster VS, ferral” to physical therapy in the literature is substantial. Self-referral has been shown to extend healthcare users’ choice of providers [17], reduce treatment delay [18], enhance patient satisfaction, and improve resources utilization efficiency [7]. Several studies have also reported that self-referral is associated with lower costs to patients, insurance providers, and healthcare organizations [8, 9, 19–21]. Although this evidence supports that self-referral is safe, efficient, and cost-effective - which indicates that it is the superior pathway- studies have reported that patients utilization of self-referral remains low [7, 8, 11].

The limited evidence surrounding self-referral service users’ actual perspectives/views for such a pathway emphasizes the need to understand how patients with musculoskeletal complaints choose a treatment pathway when seeking medical care. Our main research question was “What influences healthcare workers’ choice of treatment pathway (provider referral or self-referral) when seeking treatment for musculoskeletal complaints?”. We studied patients with musculoskeletal complaints who worked for a large, self-funded employer that reduced the cost of physical therapy services to financially promote ‘early physical therapy’ utilization in South Carolina, a state which has legislation supporting patients self-referral. By understanding the factors that influence a healthcare worker’s decision on how to access physical therapy services using qualitative research contextual evidence for the designing and re-evaluation of clinical practices may be provided.

## Methods

This was a qualitative research study involving semi-structured interviews of healthcare workers of a large self-funded employer (> 14,000 employees) in South Carolina. In 2012, the musculoskeletal program (MSK program) - a partnership between a private physical therapy organization Prisma Health [*employer*], Steadman Hawkins Clinics of the Carolinas, and Blue Cross Blue Shield [*insurance provider for all Prisma Health employees*] - encouraged its employees to choose initiating physical therapy through self-referral with a reduced patient liability ($20 copay) or the traditional provider referral pathway ($60 copay for consulting a primary care physician or $80 copay for a consulting a specialist). The program’s goal was to remove institutional barriers and give employees lower co-payments for physical therapy visits if they chose to participate. The program was marketed via department meetings, emails, and fliers.

Our study design complied with the Standards for Reporting Qualitative Research (SRQR) checklist. Purposive criterion-based sampling technique was used; the selection of participants was based on eligibility criteria that are of importance to this study [22]. Individuals were eligible to participate if they were current employees of the health system; were experiencing a spine, shoulder, knee or hip-related complaint; were participating in the MSK program; and were over the age of 18 years. Sampling was continued until saturation was reached, that is when additional interviews reveal no new information, further coding was no longer possible [23], and enough information was available to duplicate the study [24]. Patients were recruited through flyers placed in seven physical therapy clinics across a 3-county region in the southeastern metropolitan area of Greenville, South Carolina. Interested participants contacted the author by phone and were screened for eligibility before interviews were scheduled. All interviews took place in a private office between August 2017 and March 2018 and were audio-recorded. Each interview lasted approximately 40 minutes. Written informed consent was obtained, and the rights of subjects were protected. The study received ethical approval from the Prisma Health Upstate Institutional Review Board.

A sociodemographic questionnaire was administered at the end of the interview to collect information about educational attainment, ethnicity, and income. Patients’ ages and gender were abstracted from electronic health records. The Mann-Whitney U test was used to analyze continuous variables and the Fisher exact test to analyze categorical variables.

To ensure credible findings, a number of steps were taken. Two additional coders were involved to improve the credibility of the findings. Both coders understood the practice of coding and analyzed data independently to dispel any misinterpretations. The researcher along with the two additional coders met on a regular basis to discuss the codes. Each coder was allowed to create emergent codes if they saw fit. This approach ensured reliability, as well as improved the trustworthiness of the analysis. In addition, coding and preliminary findings were continually shared with two co-authors (MC and MM) as an additional check on validity.

### Semi-structured interview

Two interview guides were created—one for self-referred patients and one for provider referral patients. The interview guides (see Supplementary Material) were designed to reveal general and specific reasons why patients chose to access care in the way they did. Although both guides contained the same key questions to allow for comparison between the two groups, they differed in their follow-up questions; for instance, provider referral pathway questions probed interviewees about physician treatment recommendations and duration of use.

### Data analysis

Audio recordings of interviews were transcribed by a transcription service. To ensure privacy and confidentiality, numerical codes identified participants’ transcripts. Data collection and analysis occurred concurrently and iteratively using a grounded theory approach. This strategy was used to achieve a fine-grained understanding of how patients selected a particular treatment pathway when seeking care for their condition [25]. 1st, the research team independently conducted open, line-by-line inductive coding of transcripts [26]. Upon completion of coding, similar codes were combined, identified, and assigned conceptual labels. We applied a constant comparative method to categorize and compare data [25]  and codes were grouped into central themes [27].

## Results

**Fig. 1:**
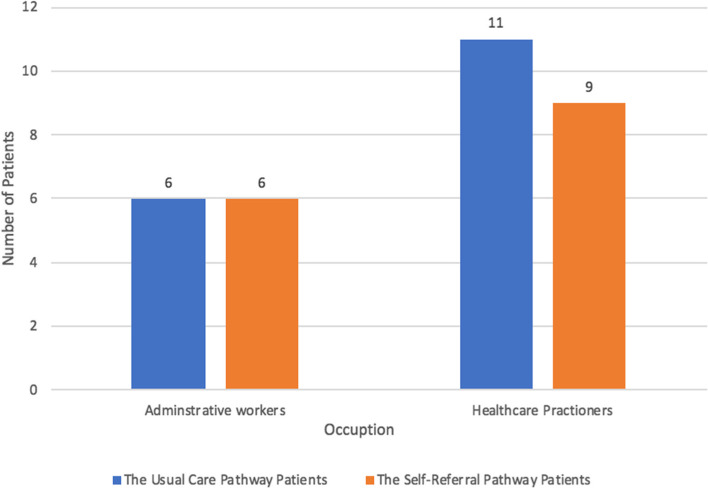
Distribution of participants occupation and their choice of treatment pathway

Thirty-two interviewees (17 interviewees were provider referred and 15 were self-referred) were enrolled in the study. A total of 20 interviewees were health care practitioners (e.g., nurses, surgical technologists, pharmacists, and respiratory therapists); the remaining 12 were administrative workers (clinical supervisors, medical transcriptions, unit secretaries, and front desk specialists). The number of healthcare practitioners and administrative workers in the two groups is presented in Fig. 1**.** Most participants in both groups complained of chronic musculoskeletal complaints. Neck, shoulder, back, and knee complaints were prevalent in both groups. Table 1 details the sociodemographic characteristics of interviewees, which did not differ between groups.

**Table 1:** Summary of the sociodemographic characteristics of patients who used the provider referral and the self-referral pathways

Variable	Choice of Treatment Pathway		*P* value
	Provider Referral Pathway Patients (*n* = 17)	Self-Referral Pathway Patients (*n* = 15)	
	n (%)	n (%)	
Age ^a^	48 (12)	51 (10)	0.306†
Gender			0.658‡
Female	13 (76)	13 (87)	
Male	4 (24)	2 (13)	
Education Attainment			0.324‡
Completed some high school	0	1 (7)	
High school graduate	3 (18)	2 (13)	
Associate’s degree	7 (41)	2 (13)	
Bachelor’s degree	5 (29)	7 (47)	
Master’s degree	2 (12)	3 (20)	
Race/Ethnicity			0.402‡
White	12 (71)	13 (87)	
Black or African American	5 (29)	2 (13)	
Hispanic or Latino	0	0	
Annual Household Income			0.885‡
Less than $24,999	1 (6)	0	
$25,000 to $49,999	4 (24)	2 (14)	
$50,000 to $99,999	8 (47)	8 (57)	
$100,000 or more	4 (24)	4 (29)	

*Note:* Income percentages for the self-referral group do not sum to 100 because of a missing value

^a^Age is a continuous variable; the values shown are means and standard deviations

† Mann-Whitney U test

‡ Fisher’s exact test

### Patients choice of treatment pathway

Before deciding to access medical care, all interviewees took self-care measures to relieve pain including analgesics and relaxation techniques. When such measures failed to provide relief, interviewees considered seeking medical care.

### The decision to use the self-referral pathway

As seen in Table 2, decisions to use self-referral rather than provider referral were driven by several factors, including knowledge of the direct access program, patients’ attitudes toward and beliefs about treatment, and previous experiences with physical therapy.

**Table 2:** Major themes and sub-themes for interviews with self-referral participants (*n* = 15)

**Theme1: Knowledge of the Direct Access Program**	
*“So, I ran into [a physical therapist]. She was in the break room the morning I was hurting so severely, and she told me to try physical therapy first to see if that would help it, and if not, they would recommend maybe a physician for me to see. At that point I figured anything would help. I felt like especially the hands on would break it because it felt like a trigger point area. I figured that was the best route to go before pursuing the surgeons or sports medicine physicians here.”* (3)	
**Theme 2: Patients’ Attitudes and Beliefs Towards Treatment**	
*Sub-Theme 1: Openness to Alternative Therapies* *“I’ve been a nurse for almost 40 years, so I’m into kind of treating myself and I think that other modalities may work first. Like heat therapy, maybe doing yoga, doing self-care treatments first.” *(23)	
*Sub-Theme 2: Beliefs About Medications and Surgery* *“The medication that I take for this [shoulder pain], because they’re not every day, makes me kind of sleepy and dull and that’s difficult to work, so I started exploring other options.” *(15) *“I wanted something that would fix it, as opposed to just tolerating it.” *(7)	
*Sub-Theme 3: Expectation of Physician Treatment Recommendation* *“So one of the reasons why I didn’t go to a physician first is just because if my pain was the result of an injury, I fell, or I was in an accident, I feel like you need the reassurance of a physician to make sure that there’s nothing broken, or that would need surgery, or something like that, but if you’re just having chronic pain that’s been coming on for a long time, my hip hurts, my knee hurts, I feel like physical therapy and exercise is most often what the treatment that’s going to be recommended, so I just went there first.” *(27)	
*Sub-Theme 4: Preference for Lower Cost Options* *“Well, I’m not going to go to the orthopedic doctor if I can go to therapy. Bypass that $50 co-pay at orthopedics and go on to therapy and see if they can help”* (32)	
**Theme 3: Resonant Prior Experience with Physical Therapy**	
*“Just due to my past experience with the results that I had with physical therapy once [a physical therapist] saw me for my lower back I went on my way. Then, I guess it was something earlier this year. I called [him] up and said, ‘I can curl, but I can’t push’. He said, ‘It sounds like a cervical issue.’ He treated me and did a great job on that.”* (6)	

Most interviewees selecting to self-refer to physical therapy knew about it through announcements and flyers distributed throughout the health system. Others learned about it from their coworkers. It was clear that these interviewees understood they could self-refer to physical therapy without seeing a physician first. However, two interviewees mentioned that knowing about the program resulted from being redirected to physical therapy when physician care was inaccessible, and they were in need for urgent medical interference because of severe pain. An example quote appears in Table 2.

Another broad theme that emerged among self-referral patients was their attitudes toward and beliefs about treatment. Four subthemes emerged: openness to alternative therapies, beliefs about medications and surgery, expectations of physicians’ treatment recommendations, and preference for lower-cost options. Quotations supporting these sub-themes appear in Table 2.

In general, patients have plenty of options for treatment of musculoskeletal complaints, and most self-referred interviewees displayed openness to a wide variety of alternative treatments and interventions. In conjunction with using medications, interviewees talked about experimenting with different modalities and providers including chiropractic care, massage therapy, and yoga to find out what works for them before seeking physical therapy.

More than half of the self-referred interviewees complained of chronic musculoskeletal pain; half of those had consulted physicians previously, which in most cases resulted in medication prescription (e.g., non-steroidal anti-inflammatory drugs). Some interviewees used these medications every time they had a flare-up; others relied on over-the-counter painkillers. Regardless of the medications involved, most interviewees expressed aversion to their use. For some interviewees, this aversion was due to experience, perceiving medications as ineffective in controlling their pain or offering long-term relief. Conversely, several interviewees acknowledged that pharmacological treatments were a moderately effective means of controlling their pain yet had concerns over their sustainability in terms of long-term and short-term side effects. This compelled them to reconsider frequent use of medications and pursue alternative treatment approaches*.* For some interviewees, it was clear that although pharmacological treatments could play a role in controlling pain, they do not offer what they want, specifically addressing the root cause of the problem rather than masking the symptoms. A few interviewees expressed concerns about needing surgery to control their pain. Awareness about the risks and complications inherent in surgical procedures drove them to seek physical therapy as a preventative measure.

Furthermore, several interviewees who self-referred to physical therapy anticipated how their treatment would unfold if they consulted a physician first. Some expected their physician to recommend physical therapy, and therefore self-referring to physical therapy was a shorter route to care. Others feared that physician consultation would result in medication prescription, which did not align with their beliefs about medications, and thus they decided to bypass the middleman and access physical therapy directly. One interviewee talked about her ability to self-diagnose and distinguish chronic pain from pain resulting from an injury or that would require surgery. She was confident that physical therapy would be recommended and therefore decided to seek physical therapy first. Among interviewees in this group, the cost associated with consulting a physician first was a driver for bypassing physician care and seeking the less costly and effective direct treatment pathway*.*

The third major theme interviewees discussed was the positive experiences they had with physical therapy. These experiences installed a belief about the efficacy of physical therapy in treating musculoskeletal complaints and substantially influenced interviewees’ choice to seek physical therapy directly for their new complaint. For these interviewees, an existing satisfactory relationship with physical therapy encouraged them to make an active choice and resort to physical therapy upon experiencing a recurrent or new pain. Other interviewees decisions to self-refer came from having clinical knowledge of physical therapy scope of practice—mainly due to being healthcare professionals—and from being exposed to their own patients’ experience with physical therapy. Many interviewees also talked about their co-workers’ experiences with physical therapy (indirect experience) and how it influenced them to bypass physician care and seek physical therapy for their musculoskeletal complaints. Quotations show in Table 2.

### The decision to use the provider referral pathway

As seen in Table 3, decisions to use the provider referral pathway were driven by lack of knowledge of the direct access program, patients’ attitudes and beliefs toward treatment and illness, and necessary physician care.

**Table 3:** Major themes and sub-themes for interviews with provider referral participants (n = 17)

**Theme1: Lack of Knowledge of the Direct Access Program**	
*“He′s the one [physician] that suggested I try this program [MSK program]. I didn’t know anything about it, and I was like, “Yeah, *$*20, that’s good. “ *(14)	
**Theme 2: Patients’ Attitudes Toward and Beliefs about Treatment and Illness**	
*Sub-Theme 1: Lack of Consideration of Physical Therapy Regardless of Past Experience* *“I don’t know. I just didn’t think about that. I really didn’t. I didn’t think physical therapy was going to help me. Because it was something, I thought I had to live with. I never thought about it, really. I didn’t want to kind of miss things and I really didn’t think about physical therapy during that part.” *(28)	
*Sub-Theme 2: Preference for Pharmacological Treatments* *“I was trying to find a quick release for the pain, instead of thinking that it was going to go away, like maybe a shot or something. I think I was looking for a quick relief when I knew it was going to take longer then, I wanted it to be over with.” *(17)	
*Sub-Theme 3: Need for Physician Reassurance* *“I wanted to make sure that there wasn’t anything structurally wrong or that there wasn’t an actual injury before starting physical therapy. I wanted the opinion of a medical doctor before I started seeking other professional consults. I didn’t want to postpone getting to the real root of the cause. If that was the case, why go to physical therapy before going to a medical doctor. I wanted to make sure that there wasn’t anything physically wrong with my shoulders.” *(4)	
*Sub-Theme 4: Coincidental Discussion* *“It was more of a convenience thing as well because I knew I had the appointment for my annual physical. I knew that if there was something that needed to be done, then he could get the ball rolling” *(25)	
**Theme 3: Necessary Physician Care**	
*“I don’t know, like I lifted something wrong or bent wrong, something along those lines. The sciatic issues that I had I had never treated medically. It was just some strengthening that relieved my problems. It probably happened two times before, not severe though. Over the next several days up until that Wednesday night that I worked I noticed my leg was starting to get painful, the sciatic type pain, the pain was just getting so severe that I ended up going to [emergency care] to see if they could do something for me.” *(2)	

One often cited theme to emerge from the provider referral patients was lack of awareness of the direct access program. Following a referral, patients were informed about the program either by their physicians or from accessing the physical therapy practice*.* While most lacked knowledge about the program, a few interviewees demonstrated knowledge yet still sought care through the provider referral pathway. For some, this was because they failed to recall the program when deciding to seek medical care for their complaint. Others lacked clarity about how the program was administered and had misconceptions about how the program worked. For instance, some interviewees assumed that a physician referral was required to receive physical therapy treatments and for insurance to cover the cost of their visits. Other interviewees, who had previously participated in the program but still opted for care through the provider referral pathway, demonstrated a lack of awareness of the ability to go straight to physical therapy. For these patients, previous participation did not create an understanding of their ability to self-refer to physical therapy for a new complaint. An example quote appears in Table 3.

Another broad theme that emerged among the provider referral was their attitudes and beliefs toward treatment and illness. Four subthemes emerged: lack of consideration of physical therapy regardless of past experience, preference for pharmacological treatments, need for physician reassurance, and coincidental discussion. Quotations supporting these sub-themes appear in Table 3.

Some interviewees who selected the provider referral pathway, despite prior experience, revealed that it had not occurred to them to consider physical therapy as a treatment option upon deciding to seek medical care. Other interviewees had a strong preference for pharmacological treatments, which they believed would provide them with an immediate solution to controlling pain. Also, a few interviewees perceptions of medication appeared to influence their preference; for instance, one interviewee, a nurse, referred to a steroid medication as a conservative treatment and preferred it over physical therapy despite positive experience. In contrast, two interviewees whose physicians suggested pharmacological treatments as first-line solutions reported strong aversion toward them. One interviewee spoke about her inclination to treat the cause of the problem rather than alleviate the symptoms. The other rejected the idea of being dependent on medications as a means to achieve relief. These interviewees reported that referral to physical therapy resulted from explicitly asking their physician to suggest an alternative treatment approach.

Moreover, interviewees using the provider referral pathway perceived physicians as experts who can make an informed clinical judgment and offer expert care. Although most of these interviewees were healthcare professionals who knew about the role of physical therapy, they clearly regarded physical therapists as non-specialists who would be unable to identify the root cause of the problem. This perception reflected their need for physician reassurance, particularly when they had concerns surrounding their illness and/or treatment such as the presence of a serious injury or an underlying medical condition. For these interviewees, physician care was the only route to gain reassurance and reach an appropriate treatment decision. Physician reassurance was also important in verifying whether physical therapy was the only available treatment option, particularly when a previous experience with physical therapy for the same pain was perceived as unhelpful or yielding only temporary results.

Although the provider referral interviewees described their pain as impairing their usual daily activities (e.g., “I could hardly even stand up” (10), “my mobility was limited” (25), they did not discuss their pain with a physician until they visited for different, unrelated complaint. It was then that they were referred to physical therapy. Some interviewees cited convenience of access as a reason for waiting on discussing their pain during their annual checkup. Others talked about their ability to tolerate pain and go on with life without seeking medical interference especially when it was of a bearable intensity.

The third major theme interviewees discussed was necessary physician care. A few interviewees sought physician care when they experienced a health crisis that dictated urgent medical attention. For instance, one interviewee spoke about his neuromuscular pain and how it would normally resolve itself within a matter of days without any medical interference; however, upon experiencing a sudden bout of intense pain, he was obliged to seek immediate care. Another interviewee also stated that her inability to recognize or pinpoint the cause of the pain or condition led her to seek physician care first. Quotations show in Table 3.

## Discussion

**Fig. 2:**
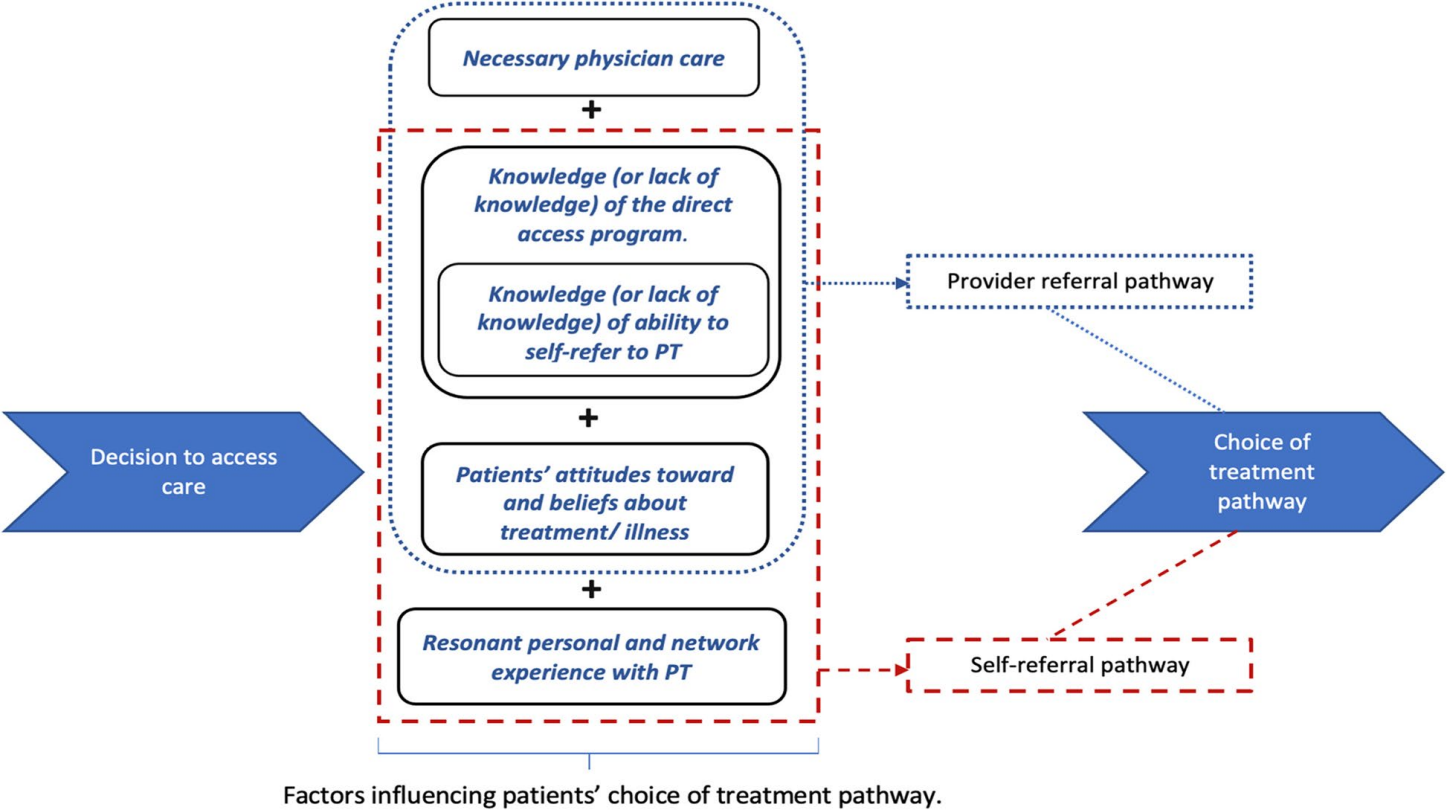
Conceptual model of patients’ choice of treatment pathway

This study has provided insights into the underlying factors that affect the choice of treatment pathway (usual care vs. self-referral) for physical therapy among healthcare workers. Based on the major themes observed, a conceptual framework showing the major factors influencing patients selecting self-referral and provider referral is shown in Fig. 2.

In this study, although self-referral to physical therapy was supported through a reduction in co-payments, its effects appear limited given the 10–15% participation rate [8]. we have found that patients’ decisions to self-refer rather than consult a medical provider were driven by an interplay between knowledge of the self-referral program, resonant prior experience with physical therapy, and patients’ attitudes and beliefs toward treatment. Knowledge of the program, especially knowledge of the ability to self-refer, was a major factor for why patients stated they did so. Consistent with previous research, in our study knowledge was a fundamental factor influencing contact initiation; lack of knowledge hinders autonomous behavior [13, 28, 29]. For instance, misconceptions surrounding the administration of the program and insurance coverage resulted in some patients seeking the provider referral pathway despite knowledge of the program. Moreover, some patients who had previously participated in the program did not consider self-referring to physical therapy initially and took the provider referral pathway for their new complaint. When questioned, patients reported a lack of knowledge of the ability to self-refer. This suggests that patients knowledge of the existence of the program does not necessarily translate into their understanding that the program allows self-referral to physical therapy. If patients’ uptake of the self-referral pathway is to be encouraged, developing effective communication strategies about patients’ ability to self-refer and how access can be achieved is of paramount.

Another strong determinant of the choice of the self-referral pathway was patients experience with physical therapy, in line with previous studies [13, 14]. Interestingly, however, our study revealed that some provider-referred patients had experience with physical therapy yet still sought physician care first. It could be, as proposed by other authors, that participants were not exposed to all physical therapy aspects resulting in insufficient overall knowledge [16]. A further reason for this finding could be that these patients past physical therapy experience was not relevant enough to encourage them to think about physical therapy for their current musculoskeletal complaint. It should be noted that provider-referred patients only talked about their previous physical therapy experience when they were specifically asked about it, unlike the self-referred patients who spoke about it spontaneously in the interviews.

Another factor influencing the selection of self-referral or provider-referral to physical therapy was attitudes toward and beliefs about treatment, in line with previous work [16]. Self-referral patients held beliefs that physical therapy and other active treatments could help in relieving their pain. While it is known that self-referred patients tend to display autonomous and proactive behavior [15, 16], we have found that this is likely related to their self-expressed aversion toward medications and the desire to treat the root cause of the problem rather than the symptoms.

However, it should be pointed out that some self-referred patients had previously received assurances from a physician that physical therapy could assist with their pain, so their behavior to access physical therapy at a later point in time was not completely autonomous. In contrast, those selecting the provider referral pathway showed a preference for physician-focused diagnosis and treatment. Specifically, they had a desire for pharmacological treatments because these treatments offered a spontaneous solution to their pain. Although prescribed medications have potential benefits in managing musculoskeletal complaints, it has been shown they may be associated with a broad spectrum of adverse effects on the gastrointestinal, cardiovascular, cerebrovascular, and renal systems [30–32].

The provider-referred patients in this study knew about the role of physical therapy in pain management yet they still chose to see a physician first. This is because they believed that physicians were more qualified to make medical judgments than physical therapists. While this finding is perhaps understandable it shows that patients acceptance of physical therapist appropriateness to make medical decisions presents a unique challenge that needs to be addressed. Given the provider referral treatment pathway implications on costs and healthcare utilization [33], strategies aimed at reshaping beliefs about the role of physical therapy, raising awareness of PT,and increasing confidence in its effectiveness both directly to the public and through partnerships with physicians, will be vital.

## Limitations, and implications for future research

This study should be interpreted within the limitations of the design. First, South Carolina does allow direct access to physical therapy (with some provisions); and the results may not apply to states with more restrictions on direct access. Recruitment involved self-selection, so patients who chose not to participate might have different reasons underlying their choice of pathway than the interviewed population. While the large female bias of the sample represented the demographics of the employer with an incentivized self-referral; a study of a more general population might generate different results. Finally, the interviews reflect the perspectives of healthcare workers of one health system in one metropolitan area within the southeastern U.S., which may limit generalizability to other settings.

## Conclusion

Healthcare workers with musculoskeletal complaints interviewed in this study based their choice of self- or provider referral pathway on their knowledge of the program, most importantly ability to self-refer, prior experiences with physical therapy, and their attitudes toward and beliefs about musculoskeletal medical treatment. Knowledge alone about physical therapy as a musculoskeletal complaint treatment option did not appear to influence healthcare workers’ choice of treatment pathway. Self-referred patients made conscious choices, attaching greater importance to their previous experience with physical therapy, while provider-referred patients’ choices were mainly determined by their attitudes and beliefs toward treatment and illness. The results of this study can be utilized to inform and guide policy makers efforts aimed at changing patients’ behaviors toward using physical therapy as a first-line approach for treating and managing their pain. It also provides physical therapists and health plan administrators with an understanding of how to empower patients to adopt autonomous health-seeking behaviors.

## Data Availability

The datasets generated and/or analysed during the current study are not publicly available due to the confidentiality and privacy reasons but are available from the corresponding author (NA) on reasonable request.

## References

[1] Sun E, Moshfegh J, Rishel CA, Cook CE, Goode AP, George SZ (2018). Association of early physical therapy with long-term opioid use among opioid-naive patients with musculoskeletal pain. JAMA Netw Open.

[2] Fritz JM, Childs JD, Wainner RS, Flynn TW (2012). Primary care referral of patients with low back pain to physical therapy: impact on future health care utilization and costs. Spine..

[3] Holdsworth LK, Webster VS, McFadyen AK (2006). The scottish physiotherapy self referral study group. self-referral to physiotherapy: deprivation and geographical setting: is there a relationship? results of a national trial. Physiotherapy..

[4] Massey BF (2002). 2002 APTA presidential address: What’s all the fuss about direct access?. Phys Ther.

[5] Moore JH, McMillian DJ, Rosenthal MD, Weishaar MD (2005). Risk determination for patients with direct access to physical therapy in military health care facilities. J Orthop Sports Phys Ther.

[6] Stokes E, Kruger J. World Confederation for Physical Therapy. Direct access and patient self-referral. LondonUK WCPT. Published online 2011. http://www.wcpt.org/sites/wcpt.org/files/files/Keynote_DirectAccess.pdf.

[7] Boissonnault WG, Lovely K (2016). Hospital-based outpatient direct access to physical therapist services: current status in Wisconsin. Phys Ther.

[8] Denninger TR, Cook CE, Chapman CG, McHenry T, Thigpen CA (2017). The influence of patient choice of first provider on costs and outcomes: analysis from a physical therapy patient registry. J Orthop Sports Phys Ther.

[9] Pendergast J, Kliethermes SA, Freburger JK, Duffy PA (2012). A comparison of health care use for physician-referred and self-referred episodes of outpatient physical therapy. Health Serv Res.

[10] Gardner K. FAQ: Direct Access at the State Level. Accessed June 14, 2017. http://www.apta.org/StateIssues/DirectAccess/FAQs/

[11] McCallum CA, DiAngelis T (2012). Direct access: factors that affect physical therapist practice in the state of Ohio. Phys Ther.

[12] Ojha HA, Snyder RS, Davenport TE (2014). Direct access compared with referred physical therapy episodes of care: a systematic review. Phys Ther.

[13] Leemrijse CJ, Swinkels ICS, Veenhof C (2008). Direct access to physical therapy in the Netherlands: results from the first year in community-based physical therapy. Phys Ther.

[14] Scheele J, Vijfvinkel F, Rigter M (2014). Direct access to physical therapy for patients with low back pain in the Netherlands: prevalence and predictors. Phys Ther.

[15] Holdsworth LK, Webster VS (2004). Direct access to physiotherapy in primary care: now?—and into the future?. Physiotherapy..

[16] Webster VS, Holdsworth LK, McFadyen AK, Little H (2008). Self-referral, access and physiotherapy: patients’ knowledge and attitudes—results of a national trial. Physiotherapy..

[17] Snow BL, Shamus E, Hill C (2001). Physical therapy as primary health care: public perceptions. J Allied Health.

[18] Durant TL, Lord LJ, Domholdt E (1989). Outpatient views on direct access to physical therapy in Indiana. Phys Ther.

[19] Hackett GI, Bundred P, Hutton JL, O’Brien J, Stanley IM (1993). Management of joint and soft tissue injuries in three general practices: value of on-site physiotherapy. Br J Gen Pract.

[20] Holdsworth LK, Webster VS, McFadyen AK (2007). What are the costs to NHS Scotland of self-referral to physiotherapy?. Physiotherapy.

[21] Mitchell JM, de Lissovoy G (1997). A comparison of resource use and cost in direct access versus physician referral episodes of physical therapy. Phys Ther.

[22] Patton MQ (2002). Qualitative Research & Evaluation Methods.

[23] Guest G, Bunce A, Johnson L (2006). How many interviews are enough?: an experiment with data saturation and variability. Field Methods.

[24] O’Reilly M, Parker N (2013). ‘Unsatisfactory saturation’: a critical exploration of the notion of saturated sample sizes in qualitative research. Qual Res.

[25] Glaser BG (1998). Doing grounded theory: issues & discussion.

[26] Stern PN (1980). Grounded theory methodology: its uses and processes. Image (IN).

[27] Glaser BG, Strauss AL. Discovery of Grounded Theory: Strategies for Qualitative Research.; 2017. Accessed December 10, 2019. https://www.taylorfrancis.com/books/e/9780203793206

[28] Andersen RM (1995). Revisiting the behavioral model and access to medical care: does it matter?. J Health Soc Behav.

[29] Kulu-Glasgow I, Delnoij D, de Bakker D (1998). Self-referral in a gatekeeping system: patients’ reasons for skipping the general-practitioner. Health Policy.

[30] Hatt KM, Vijapura A, Maitin IB, Cruz E (2018). Safety considerations in prescription of NSAIDs for musculoskeletal pain: a narrative review. PM R.

[31] Elder NC (1991). Abuse of skeletal muscle relaxants. Am Fam Physician.

[32] Ong CKS, Lirk P, Tan CH, Seymour RA (2007). An evidence-based update on nonsteroidal anti-inflammatory drugs. Clin Med Res.

[33] Childs JD, Fritz JM, Wu SS (2015). Implications of early and guideline adherent physical therapy for low back pain on utilization and costs. BMC Health Serv Res.

